# Assessment of Planting Method and Deficit Irrigation Impacts on Physio-Morphology, Grain Yield and Water Use Efficiency of Maize (*Zea mays* L.) on Vertisols of Semi-Arid Tropics

**DOI:** 10.3390/plants10061094

**Published:** 2021-05-29

**Authors:** Hanamant M. Halli, Sanganabasappa Angadi, Aravind Kumar, Prabhu Govindasamy, Raghavendra Madar, David Chella Baskar V, Hosam O. Elansary, Nissren Tamam, Ashraf M. M. Abdelbacki, Shaimaa A. M. Abdelmohsen

**Affiliations:** 1 Division of Seed Technology, ICAR-Indian Grassland and Fodder Research Institute, Jhansi 284 003, UP, India; 2 Department of Agronomy, University of Agricultural Sciences, Dharwad 580 005, KA, India; angadiss@uasd.in (S.A.); bnakumar@gmail.com (A.K.); 3 Division of Crop Production, ICAR-Indian Grassland and Fodder Research Institute, Jhansi 284 003, UP, India; prabmanikandan@gmail.com; 4 Division of Crop Production, ICAR-Indian Institute of Soybean Research, Indore 452 001, MP, India; raghavendra4449@gmail.com; 5 Agricultural Economics, College of Agriculture, Rani Lakshmi Bai Central Agricultural University, Jhansi 284 003, UP, India; davidbaskar@gmail.com; 6 Plant Production Department, College of Food and Agriculture Sciences, King Saud University, Riyadh 11451, Saudi Arabia; 7 Floriculture, Ornamental Horticulture, and Garden Design Department, Faculty of Agriculture (El-Shatby), Alexandria University, Alexandria 21545, Egypt; 8 Department of Geography, Environmental Management, and Energy Studies, APK Campus, University of Johannesburg, Johannesburg 2006, South Africa; 9 Physics Department, Faculty of Science, Princess Nourah bint Abdulrahman University, Riyadh 84428, Saudi Arabia; nmtamam@pnu.edu.sa; 10 Plant Pathology Department, Faculty of Agriculture, Cairo University, Cairo 12613, Egypt; amaeg@hotmail.com; 11 Applied Studies and Community Service College, King Saud University, Riyadh 11451, Saudi Arabia; 12 Biophysics Department, Faculty of Science, Cairo University, Cairo 12613, Egypt; shaimaa_abdelraof@yahoo.com

**Keywords:** deficit irrigation, maize, total soluble solids, planting methods, proline

## Abstract

Agriculture in a water-limited environment is critically important for today and for the future. This research evaluates the impact of deficit irrigation in different planting methods on the physio-morphological traits, grain yield and WUE of maize (*Zea mays* L.). The experiment was carried out in 2015 and 2016, consisting of three planting methods (i.e., BBF, SNF, and DWF) and four irrigation levels (i.e., I_10D_: irrigation once in ten days, I_40_: irrigation at 40% DASM, I_50_: irrigation at 50% DASM, and I_60_: irrigation at 60% DASM). The results reveal that varying degrees of water stress due to planting methods and irrigation levels greatly influenced the maize physio-morphological traits and yield attributes. The combined effect of DWF + I_50_ benefited the maize in terms of higher leaf area, RWC, SPAD values, CGR, and LAD, followed by the SNF method at 60 DAS. As a result, DWF + I_50_ and SNF + I_50_ had higher 100 grain weight (30.5 to 31.8 g), cob weight (181.4 to 189.6 g cob^−1^) and grain yield (35.3% to 36.4%) compared to other treatments. However, the reduction in the number of irrigations (24.0%) under SNF + I_50_ resulted in a 34% water saving. Thus, under a water-limited situation in semi-arid tropics, the practice of the SNF method + I_50_ could be an alternative way to explore the physio-morphological benefits in maize.

## 1. Introduction

The freshwater demand for domestic use is growing at a more rapid rate, and about 91% of the demand is used for agriculture [1]. It was reported that cereal crops consume about 50% of the total water used for food production [2]. For example, a study estimated that the yearly total consumptive water use of rice is 221 billion cubic meters (BCM), for wheat, it is 82.7 BCM year^−1^ and for maize, it is 18.02 BCM year^−1^, in India [3]. Underground water is an essential resource for food security, supporting 40% of global irrigation [4]. Nevertheless, groundwater resources are rapidly exhausted in many agricultural areas of the world [5,6]. In northern India, the groundwater depletion is estimated at about 19.2 giga tons year^−1^ [7]. Thus, groundwater needs to be recharged through the adoption of various water conservation practices in order to meet the projected food security and farm returns.

Among cereals, maize (*Zea mays* L.) is one of the most preferred and widely cultivated crops and has a great ability to adapt to various climate and soil environments. It accounts for 36% of global food grain production, alongside rice and wheat [8]. Furthermore, a steady increase in the area of maize in irrigated and rainfed areas would contribute more to cereal production [1]. However, the spatial and temporal variability in rainfall and groundwater depletion has been a challenge for the sustainability of maize production [9,10]. Although water is a critical resource, currently about 60% to 70% of irrigation water is lost through runoff, leaching and percolation, resulting in a decrease in water use efficiency (WUE). Different agronomic practices (i.e., planting methods and irrigation scheduling) may influence maize WUE by influencing plant physiological traits and yield [10,11]. Previous studies have also shown that changes in management practices such as planting methods and the level of irrigation have influenced maize growth, water and nutrient use efficiency, and grain yield [12,13,14,15]. Therefore, appropriate agronomic management practices are required to reduce water loss and increase WUE.

In this scenario, deficit irrigation may be an option to meet the partial crop water requirements and allow plants to efficiently draw moisture from the soil [16,17]. Additionally, it aims to exploit biochemical changes in the plant systems that are triggered under water stress conditions [18]. This approach thus, plays a potential role in developing water-saving strategies for maize production in semi-arid regions [19]. Studies have reported that maize can tolerate a water deficit with no significant yield loss [20,21]. The water stress tolerance traits in maize are the number of leaves, the number of stomata on the lower leaf surface, leaf orientation, ear per plant, leaf senescence, fresh root weight and root length [10]. A study conducted in China, [22] reported that mild water deficit (50% to 60%) irrigation improved the maize root to shoot ratio (0.18), WUE (3.25 g m^−2^ mm^−1^) and grain yield (1302.5 g m^−2^) compared to irrigation at high (60% to 80%) and low (40% to 50%) soil moistures. It was indicated that an acceptable yield along with high WUE was achieved in foxtail millet under alternative irrigation with mild water stress compared to severe moisture stress [23]. The authors of that study considered that it was mainly due to the modification in the physio-morphological indices such as the leaf area, the leaf dry weight, the leaf relative water content, and the chlorophyll content as compared to severe moisture stress. Similarly, [24] found the lowest leaf RWC, chlorophyll stability index, yield, and net income under severe water stress in cotton and maize in India.

The planting method also conserves the soil moisture, increases plant water availability, improves crop growth, and yield [25,26,27]. The modified furrow method of planting resulted in a higher seed yield and a maximum WUE of black gram in the semi-arid tropics [28,29]. Studies have reported that maize planted with the ridge method recorded a greater leaf area index (~6.0), 1000 grain weight (310.4 g), yield (5.45 t ha^−1^) and WUE (1.34 kg m^−3^) compared to the flat and bed methods of planting in sandy clay loam soil [30,31]. Likewise, an increase in grain yield (6.9 to 7.09 t ha^−1^) and nitrogen uptake (183.0 to 192.8 kg ha^−1^) as a result of improved root volume (4.48 to 5.03 cm^3^ plant^−1^) and root dry weight (13.89 to 14.64 g plant^−1^) was witnessed in maize planted on shallow furrows and deep ridges and furrows compared to broad bed and furrow systems in clay soil [15,32]. Therefore, it is crucial to understand the physio-morphological changes of maize according to different planting methods and irrigation schedules to explore the mechanisms of water conservation, and to achieve maximum yield.

However, very few studies have been carried out thus far to investigate the interaction effect of planting methods and irrigation levels on physio-morphology, grain yield and WUE in summer maize under field conditions [30,33,34]. Furthermore, the common practice of frequent irrigation in deeper and wider furrows (WUE; 30% to 50%) has led to higher seasonal water consumption in maize [10,15]. The estimated global average water productivity of maize crops is 1.80 kg m^−3^, with a range of 1.1 to 2.7 kg m^−3^. As a result, there are tremendous opportunities to improve agricultural productivity with 20% to 40% less water [35]. Consequently, this study was conducted on deficit irrigation using different planting methods in the southern region of India. This region is one of the largest maize-producing regions in India and represents 13.66% of the total area and 17.6% of total production, with severe water challenges and pressure for food production [36,37]. The objective of this study was to assess the influence of planting methods and irrigation levels on the physio-morphological traits, grain yield and WUE of summer maize on vertisols of a semi-arid region.

## 2. Results and Discussion

### 2.1. Maize Physio-Morphological Parameters

The effects of planting methods and irrigation levels on leaf area, RWC, SPAD reading, and canopy temperature were measured at 60 DAS (days after sowing; maximum growth stage) and 90 DAS (physiological maturity). At 60 DAS, the interaction effect of planting methods, irrigation level and year was not significant (*p* > 0.05) for leaf area, canopy temperature, RWC, CGR, and LAD of maize (Table 1). The highest leaf area and RWC were recorded for planting methods DWF (3939 cm^2^ plant^−1^ and 80.1%, respectively) and SNF (3846 cm^2^ plant^−1^ and 77.7%, respectively), while the lowest was recorded in the BBF method (3690 cm^2^ plant^−1^ and 73.8%, respectively). The same treatments resulted in a lower canopy temperature (1.26 to 1.98 °C lower) and higher CGR (15.7 g dm^−2^ day^−1^) and LAD (65.9 days) compared to BBF (Table 1). Among irrigation levels, I_40_ and I_50_ produced a higher and comparable leaf area, CGR, and LAD (Table 1). The interaction effect of planting methods by irrigation level was significant for the SPAD reading at 60 DAS (Table 2). For irrigation levels I_40_ and I_50_, the maximum SPAD reading (57.5 and 55.9 cm^2^ plant^−1^, respectively) was recorded under planting method DWF, followed by I_40_ under SNF (Table 2). The year factor also had an effect on leaf area, RWC, canopy temperature, CGR, and LAD. Compared to 2016, a higher leaf area (3893 cm^2^ plant^−1^), RWC (77.92%), CGR, and LAD and a lower canopy temperature (30.91 °C) were reported in 2015 (Table 1). The greater moisture availability, aeration, nutrients, stomatal conductance, and free flow of CO_2_ into mesophyll cells of maize plants under DWF and SNF might have resulted in the enhanced leaf area, RWC, SPAD value, CGR, and LAD in this study. The improved soil water availability under the ridge and furrow system was an important factor for the higher maize plant height (5.86% greater) and accumulation of photosynthates (7.41%) compared to the bed and flat systems [30,38,39]. Adequate photosynthesis, plant growth and leaf retention under optimum moisture conditions also linked to a greater leaf area, SPAD value, RWC, CGR, and LAD and a lower canopy temperature in plants [40,41]. Water stress is one of the factors reducing leaves and the overall growth of plants. It can regulate many aspects such as stomata opening, chlorophyll content, carbohydrate formation, photosynthesis and plant leaf elongation and expansion. Therefore, optimal irrigation at critical stages of maize (seedling, knee-high, tasseling, and silking stages) has a considerable benefit on growth and development.

**Figure 1:**
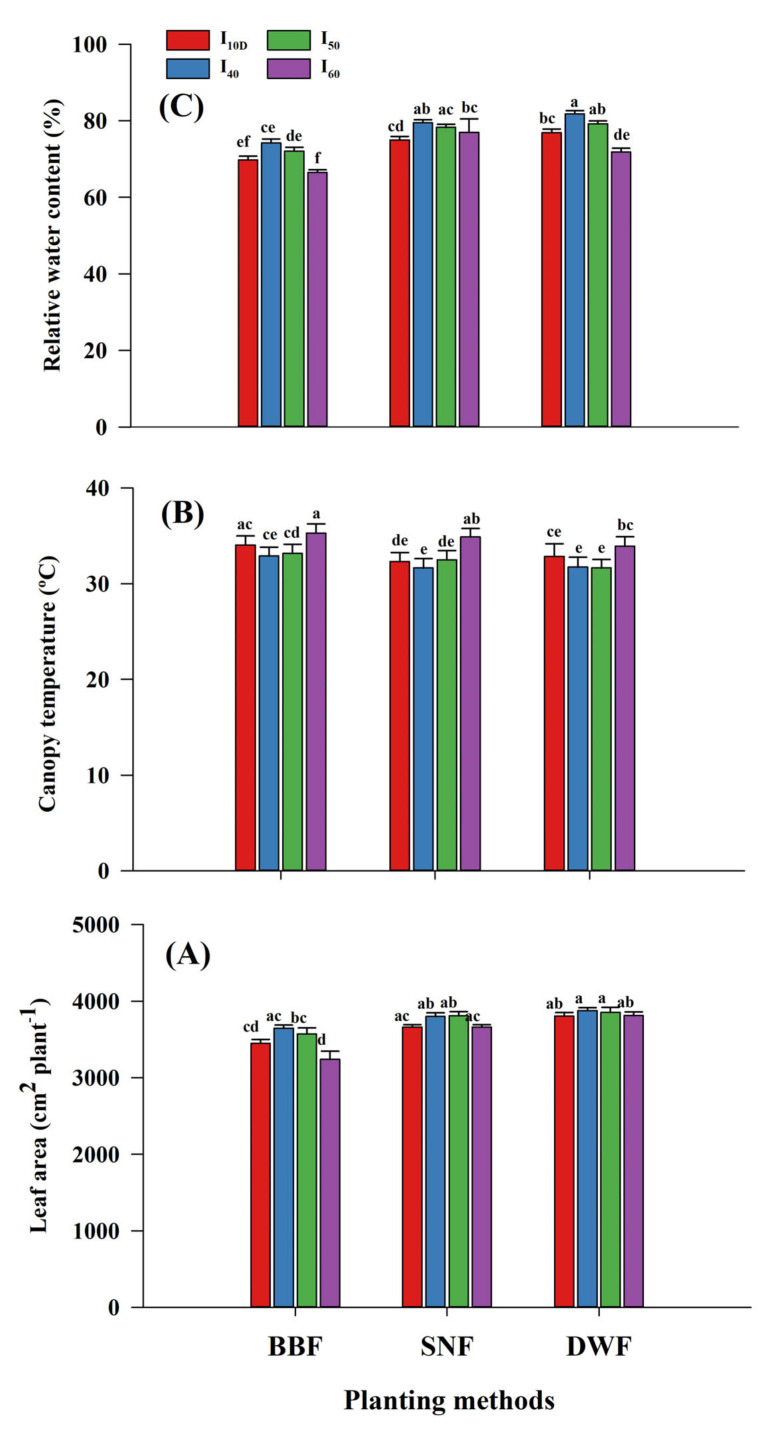
Influence of planting methods and irrigation levels on leaf area (**A**), canopy temperature (**B**) and relative water content (**C**) of maize at 90 DAS. Means followed by the same letter (s) within a bar are not significantly different.

At 90 DAS, the leaf area, canopy temperature, and RWC of maize as influenced (*p* <0.05) by interactions of planting methods by irrigation level are presented in Figure 1A–C. Among irrigation levels, I_40_ and I_50_ recorded a higher leaf area (3876 and 3852 cm^2^ plant^−1^, respectively) and RWC (81.8% and 79.2%, respectively) and a lower canopy temperature (31.26 and 32.86°C, respectively) under the DWF planting method compared to other treatment combinations. However, the interaction effect (planting methods by irrigation level) on the SPAD value at 90 DAS was not significant (*p* > 0.05) as per Table 3. The results show that a greater SPAD value was recorded with the DWF method (3.5 to 6.01 units greater) and I_40_ irrigation level (1.76 to 7.5 units greater) compared to other treatments (Table 3). The authors presumed that irrigation at lower (I_40_) and moderate (I_50_) depletion under the DWF method might improve the moisture and nutrient availability and further crop uptake due to improved soil mineralogy and microbial activity. Overall, there was a declining trend for the leaf area, SPAD value, and RWC between 60 and 90 DAS. Possibly, maize plants attained peak growth due to assimilated maximum photosynthates up to 60 DAS and later transferred towards the development of reproductive parts (flowers and cobs) as reflected in terms of the marginal reduction in the leaf area, SPAD, RWC, CGR, and LAD. However, the canopy temperature was higher at 90 DAS than at 60 DAS. With respect to the effect of year on the leaf area, canopy temperature, and RWC of maize, the results are presented in Table 4. Table 4 shows that the year 2016 recorded a lower leaf area and RWC compared to 2015, and this was primarily due to the higher mean temperature, evaporation rate, and soil temperature, as indicated by the increase in the canopy temperature. It was previously reported that as adverse weather events increased, remobilization of water from old to new leaves also increased as a result of early leaf senescence and reduced leaf area and leaf water content in maize [42].

### 2.2. Biochemical Compounds of Maize

The effects of planting methods, irrigation level and year on crude protein content, proline content, and total soluble sugar content of maize are presented in Table 5 and Table 6. Crude protein and proline contents of maize were influenced by the interaction of planting methods by irrigation level (Table 5). The BBF (23.10%) and SNF (19%) methods recorded a higher crude protein content under the I_60_ irrigation level compared to other treatment combinations. A year effect was not observed with the crude protein content of maize. The content of the non-essential amino acid “proline” was also higher in the BBF method with I_60_ (8.7%), followed by SNF with I_60_ (8%). A significant effect of year was observed with the proline content, with 2016 reporting a higher proline content (5.10% higher) than 2015. Proline acts as a membrane protector by stabilizing macromolecules, maintaining the cell turgor pressure, and as a carbon sink under moisture stress conditions. A similar result was also reported by [43], wherein higher water stress increased the crude protein content of corn grains in the sub-humid region of Turkey. The higher accumulation of crude protein was recorded in autumn-planted sugar beet roots (5.2 μ Mol g^−1^ fw) compared to spring (2.80 μ Mol v^−1^ fw) due to the higher plant temperature and moisture stress [44]. The accumulation of proline is the result of the reciprocal regulation of the pathways and the repressed catabolic pathway under oxidative stress which helps to reduce the cellular damage [45]. In addition, proline content increases in maize under water stress due to interference in protein synthesis, reduced photosynthesis, enzyme activity, and osmotic potential in the cytoplasm [43,46]. Previous studies also associated a higher amount of proline (2.54 to 3.36 times) and soluble sugars (1.60 to 1.89 times) and a lower amount of starch (51% to 58% decrease) with a depleted rate of irrigation (50% depletion) in maize [47,48]. Likewise, [44] reported that water stress increased the proline content of sugar beet (2.10 μ mol g^−1^ fw) over irrigated treatment (0.98 μ mol g^−1^ fw) in southern Spain.

Likewise, TSS content in maize grains was higher with BBF (13.0%) compared to SNF (12.07%) and DWF (11.60%) methods (Table 6). Irrigation level I_60_ had a 6.82% higher concentration of TSS over I_50_ and 9.61% over I_40_. Similar to proline, a year effect also observed with TSS content. Compared to 2015, a 3.14% higher concentration of TSS was found in 2016. Starch degradation during severe water stress may increase the concentration of TSS [49]. Water stress also induces the conversion of hexoses and other carbohydrates, such as sucrose and starch, into sugar alcohols (polyols) and proline. Therefore, the accumulation of these osmo-protectants may determine the tolerance of the plant to a water deficit condition. As a result, the current study shows that plants accumulate a higher concentration of biological compounds such as total soluble solids, proline and crude proteins under increased water stress. These compounds play an imperative role in tolerating water stress and satisfying crop water needs during drought.

### 2.3. Days to Tasseling, Silking, and Physiological Maturity in Maize

The individual effect of planting methods, irrigation level and year was significant (*p* <0.05) for days to tasseling, silking, and physiological maturity of maize (Table 6); however, the interaction effects were not significant (*p* > 0.05). Among the planting methods, BBF, at 2.89, 2.89, and 3.06 days earlier, and SNF, at 1.22, 1.22, and 1.3 days earlier, exhibited faster tasseling (50%), silking, and physiological maturity, respectively, compared to DWF. Irrigation at a higher depletion rate (I_60_) resulted in earlier tasseling (50%), silking, and physiological maturity over other irrigation treatments. The range was 57 to 59 days for 50% tasseling, 61 to 64 days for 50% silking, and 99 to 102 days for physiological maturity. With respect to years, 50% tasseling (2.85 days earlier), silking (1.85 days) and physiological maturity (2.05 days) were more rapid in the year 2016 compared to 2015. Soil moisture stress may cause plants to mature earlier as a physiological mechanism to overcome any type of stress. The diversion of photosynthates to the reproductive phase under partially water-stressed BBF and SNF and 60% depletion irrigation treatment could be the reason for the faster reproductive phase in those treatments. Furthermore, the increased soil moisture availability under DWF and irrigation at 40% and 50% depletion treatments could have prolonged the crop root forage area due to mineralization of an unavailable nutrient pool and optimum moisture content. Therefore, improved root growth and vegetative phases were witnessed under DWF + I_40_ and I_50_ [32]. The low rainfall (105.8 mm), higher average maximum temperature (35.99°C) and evaporation (7.43 mm day^−1^) and increased soil temperature (40.89°C) in 2016 (Table 7) are believed to be reasons for the early occurrence of 50% tasseling, silking, and physiological maturity compared to 2015. These results are consistent with the findings of [50], who confirmed that conventional irrigation recorded significantly longer tasseling (4.95 days) and silking (6.0 days) durations as compared to the alternative furrow irrigation method in India. Similarly, [51,52] observed early tasseling and silking in maize with irrigation intervals at 7 to 12 days. Therefore, the practices of DWF and I_40_ and I_50_ would be more beneficial to maize in terms of higher accumulation of photosynthates.

### 2.4. Effect on Yield Parameters

**Figure 2:**
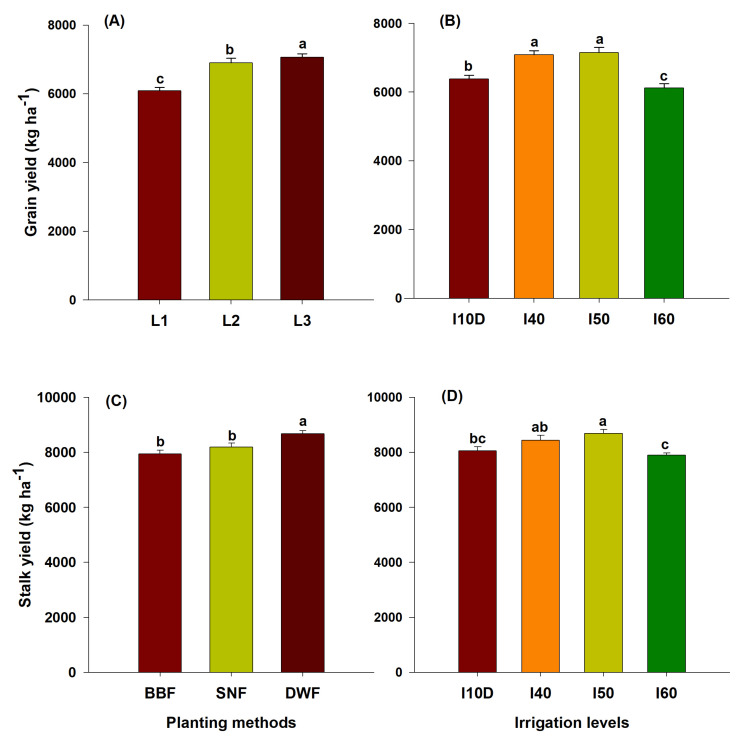
Grain (**A**,**B**) and stalk (**C**,**D**) yields of maize in response to planting methods and irrigation levels. Means followed by the same letter (s) within a column are not significantly different.

Maize yield parameters (100 grain weight and cob weight) and grain yield were influenced by the interaction of planting method by irrigation level (*p* <0.05), as summarized in Table 8 and Figure 2A,B. The planting method DWF under irrigation levels I_40_ and I_50_ recorded a higher 100 grain weight (32 and 31.80 g, respectively) and cob weight (188.6 and 189.6 g, respectively). There was an improvement in yield attributes, i.e., 100 grain weight and cob weight, under SNF and I_50_, and DWF and I_50_, which recorded 36.42% and 35.32% higher grain yield, respectively, compared to BBF and I_50_. Among the planting methods, the grain yield was 11.7% to 13.8% greater in the SNF and DWF methods than BBF (Figure 2A). The irrigation levels I_50_ (6972 kg ha^−1^) and I_40_ (6963 kg ha^−1^) recorded a higher grain yield over I_10D_ (6533 kg ha^−1^) and I_60_ (6266 kg ha^−1^) (Figure 2B). Furthermore, as expected, the year 2016 recorded the lowest 100 grain weight (0.72 g lesser), cob weight (5.4 g) and grain yield (464 kg ha^−1^) compared to 2015. A similar trend was also noted with respect to the stalk yield (Figure 2C,D). It was opined that water stress-induced floret abortion under the BBF method of planting (six irrigations less) and in the I_60_ irrigation level (328.5 mm less water) could be a reason for the lower grain weight, cob weight, and yield. Henceforth, the crop was not able to have a higher number of effective florets per flower. The water stress effect is also reflected in the result of reduced CGR, LAD, and leaf RWC (Table 1). Our experimental results are in agreement with the findings of [26,53], wherein there was a substantial grain yield reduction in maize due to aborted embryos as a result of severe moisture stress conditions. In addition, soil water stress can cause early senescence of lower leaves and result in decreased biomass accumulation and grain yield due to reduced interception of photosynthetically active radiation [54,55]. Therefore, improvement in RWC, CGR, LAD, and yield attributes, coupled with regulated bio-accumulates (i.e., proline, crude protein, and TSS), possibly resulted in a higher grain yield [15,54]. Overall, the DWF and SNF planting methods with allowable moisture depletion irrigation methods (I_40_ and I_50_) can be a viable option for higher maize grain and stalk yields under semi-arid regions.

### 2.5. Water Use Efficiency of Maize

The interaction effect of the planting method by irrigation level was not significant for the grain WUE (*p* > 0.05) as per Table 6, but it was significant for the biomass WUE (*p* <0.005), as summarized in Table 8. However, the individual effects of planting methods, irrigation levels and year were significant for both grain and biomass WUEs (*p* < 0.0001). The results show that higher grain WUE was recorded in BBF (16.38 kg ha-mm^−1^), followed by the SNF method (15.32 kg ha-mm^−1^), and the lowest was in the DWF method (12.94 kg ha-mm^−1^) (Table 6). Among irrigation levels, irrigation at I_60_ had a greater grain WUE (15.80 kg ha-mm^−1^) compared to I_10D_ (14.51 kg ha-mm^−1^), I_40_ (13.75 kg ha-mm^−1^) and I_50_ (15.46 kg ha-mm^−1^). Compared to 2016, a 13% greater grain WUE was recorded in 2015. Similarly, the biomass WUE was highest with the BBF and I_60_ (42.53 kg ha-mm^−1^) treatments, followed by BBF and I_50_ (37.80 kg ha-mm^−1^). The year 2015 (34.27 kg ha-mm^−1^) also recorded a higher biomass WUE compared to 2016 (31.76 kg ha-mm^−1^). The higher WUE in the BBF method is likely due to reduced water consumption (32.62% and 17.88% lower, Table 9) compared to DWF and SNF. Interestingly, I_50_ recorded a higher and comparable grain yield with a considerable reduction in the water consumption (12.52% to 37.92%) compared to I_40_, whereas the excess lateral moment of soil moisture under the DWF method led to a higher water consumption (30% to 35% higher irrigation) and low WUE, as reported by [32]. Similar results were obtained by [56], who reported that a greater WUE (13.63% greater) was observed with moderate-deficit irrigation compared to full irrigation in clay loam soil. The accumulation of bio-compounds such as TSS and proline build-up during the state of water stress might regulate the osmotic potential that could have improved maize WUE [47,57]. Therefore, the BBF and SNF methods with moderate irrigation (I_50_) could be a potential option under a water scarcity situation for higher WUE.

## 3. Materials and Methods

### 3.1. Study Location

The field experiment was carried out during the summer season (February to May) at the University of Agricultural Sciences research farm in Dharwad, India (15°29′20.71″ N, 74°59′3.35″ E and 678 m above mean sea level) in 2015 and 2016. Rainfall levels and intensities varied over the study period (February to May). Rainfall was 247.88 and 105.8 mm, respectively, in 2015 and 2016, and rainy days totaled 11 in 2015 and 7 in 2016. The highest maximum temperature was recorded in April (35.1 °C in 2015 and 38.0 °C in 2016), while the lowest minimum temperature was recorded in February (14.6 °C in 2015 and 17.9 °C in 2016). The average evaporation rate was greater in 2016 (7.43 mm day^−1^) as compared to 2015 (5.92 mm day^−1^). Compared to April 2015 (41.96 °C), the average soil temperature was higher (44.36 °C) in April 2016. The soil type at the experimental site was clay with a pH of 7.83 (neutral to slightly alkaline) and an electrical conductivity of 0.24 dS m^−1^ (normal). Further, the soil was medium in organic carbon content (0.62%), medium in available nitrogen (320.3 kg ha^−1^) and phosphorus (33.32 kg ha^−1^) and high in available potassium (426.5 kg ha^−1^). The other important meteorological parameters and soil properties are presented in Table 7 and Table 10, respectively.

### 3.2. Experimental Design and Field Management

**Figure 3:**
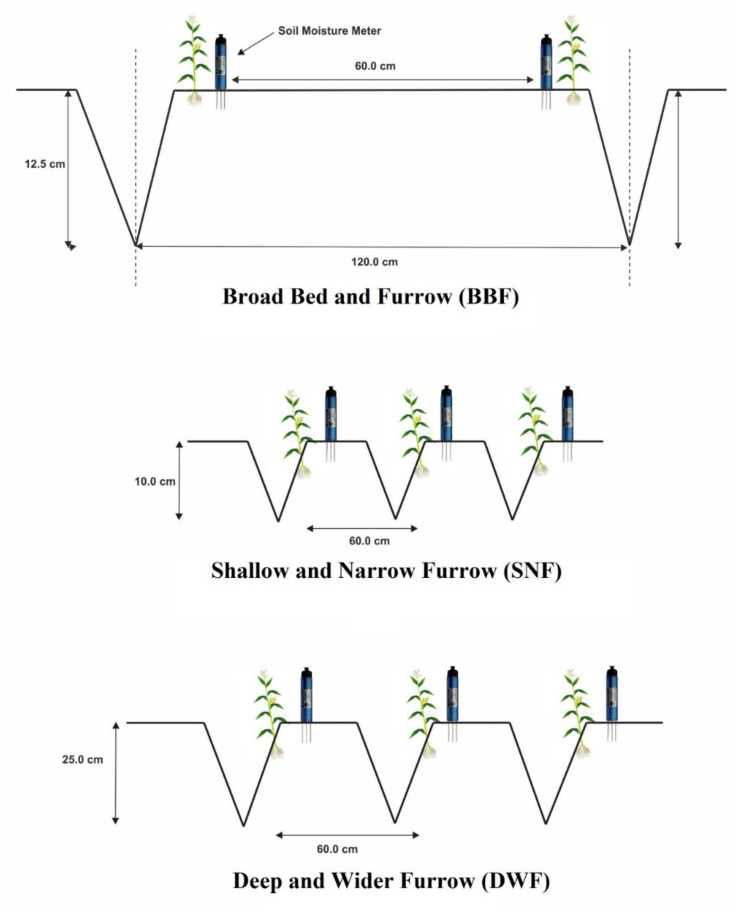
Schematic diagram of planting methods.

In this research, four irrigation levels (i.e., irrigation once in ten days (I_10D_), irrigation at 40% depletion of available soil moisture (DASM) (I_40_), irrigation at 50% DASM (I_50_), and irrigation at 60% DASM (I_60_)) were studied under three planting methods (i.e., broad bed and furrow (BBF), shallow and narrow furrow (SNF), deep and wider furrow (DWF)). The experiment was arranged in a split-plot design by keeping planting methods in main groups and irrigation levels in secondary groups, and all treatments were replicated 3 times. The main plots had a dimension of 23.2 × 7.0 m^2^ and sub-plots were 6.0 × 5.4 m^2^ in dimension. The furrow depths of 0.12 m for BBF, 0.10 m for SNF, and 0.25 m for DWF were maintained throughout the study period (Figure 3). However, a uniform plant population (833,333 plants ha^−1^) was maintained in all the planting methods. The hybrid maize “Pinnacle” (Monsanto, Hyderabad-501501, India) was planted on the side of the ridges with a spacing of 60 × 20 cm^2^ on 7 February in 2015 and 1 February in 2016. The crop was fertilized with 150 kg N (CO (NH_2_)_2_), 75 kg P_2_O_5_ (Ca (H_2_PO_4_)_2_) and 37.5 kg K_2_O (KCl) ha^−1^. Amounts of 50% of total N and 100% of P and K were applied at the time of sowing, and the remaining 50% of N was applied as a top dressing in two splits, one at 30 DAS (V9 stage) and the second at 60 DAS (tasseling stage). Weeds were managed using a pre-emergence application of atrazine (1.0 kg ai ha^−1^). The crop was harvested on 31st May in 2015 (115 days) and 21st May in 2016 (110 days).

### 3.3. Soil Moisture Measurement and Irrigation Scheduling

Irrigation was scheduled on the basis of DASM in different planting methods throughout the crop growth stages. The theta probe (MPKit-406 Soil Moisture Instant Reading Kit, ICT International, Spectra Agritec, New Delhi-110008, India), was used (rapid method) to measure soil water content. The soil samples were randomly taken between maize plants in all treatments prior to each irrigation. The probe readings were compared with a standard gravimetric method for calibration purposes. The soil water status was regularly monitored to schedule the irrigation by inserting the probe into the root zone. The field was irrigated when the respective lower limit of depletion was reached (i.e., 40%, 50%, and 60%), whereas a uniform irrigation was provided up to 20 DAS for better crop establishment in all the treatments. At each depletion point, the soil moisture content (%) was calculated using the formula provided by [58]. This method of withholding irrigation to allowable soil moisture depletion was similar to [59].

Moisture content (%) = ((FC − PWP) × Depletion (%))/100 + PWP

The discharge of bore well water was measured (4.3 L s^−1^) using a Parshall flume (throat section size 7.5 cm, Hydro Flow-Tech Engineers, India, Maharashtra-422005) with the help of a calibrated table, as suggested by [58]. Both sides of the furrow were regularly watered in SNF and DWF. However, only one side of the furrow was irrigated in the BBF method. Separate irrigation channels were prepared between the main plots to prevent lateral water movement. Based on the discharge, the time taken to irrigate, the number of irrigations and the depth of irrigation water were calculated. The amount of rainfall was also taken into account while calculating the total amount of water used (Table 9).

### 3.4. Measurement of Crop Growth and Phonological Parameters

Leaf area was recorded using a leaf area meter (LICOR-220, plant canopy analyser, Elron Instrument Company Pvt. Ltd. New Delhi-110019, India). The crop growth rate (CGR) was calculated at different intervals and expressed in g dm^−2^ day^−1^, as suggested by [60]. Likewise, leaf area duration (LAD) was calculated from the leaf area index of the crop at different intervals [61].

CGR (g dm^−2^ day^−1^) = (W_2_ − W_1_)/((t_2_ − t_1_) × (spacing))

where W_2_ and W_1_ are the plant dry weight (g) recorded at time t_2_ and t_1_ (days), respectively.

LAD (days) = ((L_i_ )+ (L_i+1_))/(2 × (t_2_ − t_1_))

where L_i_ = LAI at i stage, L_i + 1_ = LAI at i + 1st stage and t_2_ − t_1_ = time interval between L_i+1_ and L_i_ stages.

The SPAD meter (Soil Plant Analysis Development, Nunes Instruments, Coimbatore-641018, Tamil Nadu, India) reading was recorded to estimate the leaf chlorophyll content by selecting the third fully expanded leaf from the apex. To do so, several measurements were conducted on each leaf at the top and in the middle and finally averaged. Similarly, the relative water content (RWC) of the 3 fully open leaves from the top was computed using the formula provided by [62].

RWC (%) = (FW − DW)/(TW − DW) × 100

where FW—fresh weight of leaf (g); DW—dry weight (g); and TW—turgid weight (g).

The total soluble solids (°brix) content of the maize grains was measured using the Labart hand refractometer (Hand Brix Refractometer, 0–18%, X Tech Lab Supplies, New Delhi-110086, India) at the milky stage and expressed in percentage. The canopy temperature was recorded with an infra-red thermometer (GM320 non-contact laser temperature gun, −50°Cto 330°C, Macfos Pvt. Ltd., Pune-411026, India). These measurements were conducted throughout the crop growth stage on a clear day at solar noon when the angle of elevation of the sun is maximum (bright sunshine hours) and expressed in °C. The proline content in fresh leaf tissue was determined and calculated by using the following formula [63] at 520 nm in a spectrophotometer.

Proline (μ mol g^−1^)= (34.11 × OD520 × V)/(2 × f)

where V—total volume of extract; f—grams of fresh leaf; 2—volume of extract taken; and OD—optical density.

The phenological observations such as the number of days taken for 50% tasseling, silking and physiological maturity of the plants from the date of sowing were recorded from all the treatments. Maize cobs were harvested at a moisture level of approximately 13%. The grain and stalk yields were calculated from the net plot. The seeds from each plot were taken and 100 medium-sized grains were counted and weighed (g). Later, the crude protein content of maize grains was computed based on the nitrogen (N) content in the grain sample (crude protein = N × 6.25), as described by [64], and expressed in percentage.

### 3.5. Water Use Efficiency (WUE)

The treatment-wise WUE was calculated by taking the ratio of grain/biomass yield produced and total water used by the maize, as described by [22,65].

WUE (kg ha-mm^−1^) = (Kernal/biomass yield (kg ha^−1^))/(Water applied (ha-mm))

### 3.6. Statistical Analysis

ANOVA was conducted for all the variables using a mixed model (Proc GLIMMIX, SAS v 9.3. SAS Institute, Inc., Cary, NC, USA) and the treatment means were separated using Fisher’s least significance difference (LSD) test. The significance of all data was tested at α < 0.05. Prior to ANOVA, the normality of the experimental data was checked using Proc Univariate analysis (Shapiro–Wilk test) in collaboration with colleagues from King Saud and Princess Nourah bint Abdulrahman University. All data tested were normal and no transformation was required. Planting method, irrigation schedule, year and their interactions were considered as fixed effects, and replications were considered as random effects.

## 4. Summary

The water stress level has a proportional influence on the physio-morphology of crop plants. However, changes to agronomic management practices could improve resource use efficiency and crop yield. Moderate-deficit irrigation (I_50_) under the DWF and SNF methods favored the maize yield attributes by maintaining a higher growth (CGR and LAD) and internal water balance (RWC, leaf area, SPAD, proline, and TSS contents). Therefore, the I_50_ irrigation level under both DWF and SNF improved the grain WUE by 12.40% and biomass WUE by 13.7% compared to irrigation at lower depletion (I_40_). However, the SNF method used less irrigation water than the DWF method. Therefore, practicing deficit irrigation (I_50_) under the SNF method could be a better choice for a higher maize grain yield and WUE in semi-arid tropics.

## Figures and Tables

**Table 1:** Effect of planting methods and irrigation levels on leaf area, canopy temperature, leaf relative water content (RWC), crop growth rate (CGR), and leaf area duration (LAD) of maize at 60 DAS in Dharwad, India.

Treatment *		Leaf Area (cm^2^ Plant^−1^)	Canopy Temperature (°C)	RWC (%)	CGR (g dm^–^^2^ day^−1^)	LAD (Days)
Planting methods (PM)						
BBF		3690 (±70.4 **) b	33.1 (±0.41) a***	73.8 (±0.8) c	14.1 (±0.2) b	57.4 (±0.6) b
SNF		3846 (±45.1) ab	31.8 (±0.4) b	77.7 (±0.9) b	14.5 (±0.2) b	63.7 (±0.8) a
DWF		3939 (±40.4) a	31.1 (±0.3) c	80.1 (±0.8) a	15.7 (±0.3) a	65.9 (±0.7) a
Irrigation levels (IL)						
I_10D_		3879 (±48.2) ab	32.2 (±0.4) b	76.9 (±0.9) b	14.2 (±0.2) b	62.9 (±1.0) ab
I_40_		3957 (±39.2) a	31.0 (±0.3) c	80.8 (±0.9) a	15.8 (±0.2) a	65.3 (±1.0) a
I_50_		3894 (±60.1) a	30.7 (±0.2) c	79.4 (±0.7) a	15.5 (±0.2) a	63.1 (±1.1) a
I_60_		3570 (±72.4) b	34.2 (±0.4) a	71.7 (±0.8) c	13.6 (±0.2) b	58.0 (±1.3) b
Year						
2015		3893 (±38.0) a	30.9 (±0.2) b	77.9 (±0.8) a	15.0 (±0.1) a	63.5 (±0.5) a
2016		3757 (±38.0) b	33.2 (±0.2) a	76.5 (±0.8) b	14.6 (±0.1) b	61.1 (±0.5) b
Source of variation	DF	_________________________*p*-value (<0.05)__________________________				
PM	2	0.001	<0.0001	<0.0001	<0.0001	<0.0001
IL	3	<0.0001	<0.0001	<0.0001	<0.0001	<0.0001
Year	1	0.01	<0.0001	0.02	0.03	0.003
PM × IL	6	NS	NS	NS	NS	NS
PM × IL × Year	6	NS	NS	NS	NS	NS

* BBF, broad bed and furrow; SNF, shallow and narrow furrow; DWF, deep and wider furrow; I_10D_, irrigation once in 10 days; I_40_, irrigation at 40% DASM; I_50_, irrigation at 50% DASM; and I_60_, irrigation at 60% DASM. ** Standard error of mean. *** Means followed by the same letter (s) within a column are not significantly different.

**Table 2:** Interaction of planting methods, irrigation levels, and year influenced SPAD reading of maize at 60 DAS in Dharwad, India.

Treatment *		SPAD Reading
Planting Method (PM)	Irrigation Levels (IL)	
BBF	I_10D_	46.6 (±0.3 **) ef ***
	I_40_	49.6 (±1.0) cd
	I_50_	47.8 (±0.6) de
	I_60_	43.8 (±0.4) g
SNF	I_10D_	48.7 (±0.6) ce
	I_40_	53.1 (±0.5) ab
	I_50_	51.3 (±0.5) bc
	I_60_	44.7 (±0.6) fg
DWF	I_10D_	49.3 (±0.5) cd
	I_40_	57.5 (±0.6) a
	I_50_	55.9 (±0.4) a
	I_60_	49.2 (±0.5) ce
Source of variation	DF	*p*-value (<0.05)
PM	2	<0.0001
IL	3	<0.0001
Year	1	0.004
PM×IL	6	<0.0001
PM × IL × Year	6	NS

* BBF, broad bed and furrow; SNF, shallow and narrow furrow; DWF, deep and wider furrow; I_10D_, irrigation once in 10 days; I_40_, irrigation at 40% DASM; I_50_, irrigation at 50% DASM; and I_60_, irrigation at 60% DASM. ** Standard error of mean. *** Means followed by the same letter (s) within a column are not significantly different.

**Table 3:** Effect of planting methods, irrigation levels, and year on SPAD reading of maize at 90 DAS in Dharwad, India.

Treatment *		SPAD Reading
Planting methods (PM)		
BBF		47.0 (±0.5 **) c ***
SNF		49.5 (±0.7) b
DWF		53.0 (±0.8) a
*p*-value (<0.05)		<0.0001
Irrigation levels (IL)		
I_10D_		48.2 (±0.4) c
I_40_		53.4 (±0.9) a
I_50_		51.6 (±0.8) b
I_60_		45.9 (±0.6) d
*p*-value (<0.05)		0.0001
Source of variation	DF	*p*-value (<0.05)
PM	2	<0.0001
IL	3	<0.0001
Year	1	NS
PM × IL	6	NS
PM × IL × Year	6	NS

* BBF, broad bed and furrow; SNF, shallow and narrow furrow; DWF, deep and wider furrow; I_10D_, irrigation once in 10 days; I_40_, irrigation at 40% DASM; I_50_, irrigation at 50% DASM; and I_60_, irrigation at 60% DASM. ** Standard error of mean. *** Means followed by the same letter (s) within a column are not significantly different.

**Table 4:** Effect of year on leaf area, canopy temperature and leaf relative water content (RWC) of maize at 90 DAS in Dharwad, India.

Year	Leaf Area (cm^2^ Plant^−1^)	Canopy Temperature (°C)	RWC (%)
2015	3653 (±22.59 *) a**	30.94 (±0.11) b	75.64 (±0.92) a
2016	3709 (±22.59) a	35.21(±0.11) a	74.81 (±0.92) a
*p*-value	NS	<0.0001	NS

* Standard error of mean ** Means followed by the same letter (s) within a column are not significantly different.

**Table 5:** Effect of planting methods, irrigation levels and year on crude protein content (CPC) and proline content of maize at Dharwad, India.

Table *		CPC (%)	Proline (%)
Year			
2015		10.4 (±0.02 **) a	14.1 (±0.2) b ***
2016		10.3 (±0.03) a	14.8 (±0.3) a
PM × IL			
BBF	I_10D_	18.8 (±0.5) bc	6.0 (±0.6) f
	I_40_	13.4 (±0.4) gf	7.0 (±0.5) d
	I_50_	13.9 (±0.4) ef	6.0 (±0.5) f
	I_60_	23.1 (±0.3) a	8.7 (±0.4) a
SNF	I_10D_	15.8 (±0.5) de	6.5 (±0.7) e
	I_40_	10.7 (±0.4) hi	6.0 (±0.5) f
	I_50_	11.8 (±0.4) g–i	7.0 (±0.6) d
	I_60_	19.0 (±0.4) a	8.0 (±0.5) b
DWF	I_10D_	11.9 (±0.2) gh	6.5 (±0.4) e
	I_40_	8.4 (±0.4) j	5.0 (±0.5) g
	I_50_	9.9 (±0.4) ij	7.0 (±0.4) d
	I_60_	16.9 (±0.4) cd	6.5 (±0.4) e
Source of variation	DF	^______________________^ *p*-value (<0.05) ^___________________________^	
PM	2	<0.0001	<0.0001
IL	3	<0.0001	<0.0001
Year	1	NS	0.001
PM × IL	6	<0.0001	0.011
PM × IL × Year	6	NS	NS

* PM, planting methods; IL, irrigation levels; BBF, broad bed and furrow; SNF, shallow and narrow furrow; DWF, deep and wider furrow; I_10D_, irrigation once in 10 days; I_40_, irrigation at 40% DASM; I_50_, irrigation at 50% DASM; and I_60_, irrigation at 60% DASM. ** Standard error of mean. *** Means followed by the same letter (s) within a column are not significantly different.

**Table 6:** Effect of planting methods, irrigation levels and year on total soluble solids (TSS), days to 50% tasseling, days to 50% silking, days to physiological maturity, and grain WUE of maize at Dharwad, India.

Treatment *		TSS (%)	50% Tasseling (Days)	50% Silking (Days)	Physiological Maturity (Days)	Grain WUE (kg ha-mm^−1^)
Planting methods (PM)						
BBF		13.0 (±0.2 **) a	57.0 (±0.4) c	61.5 (±0.3) c	99.5 (±0.35) c	16.4 (±0.3) a ***
SNF		12.1 (±0.1) b	58.7 (±0.3) b	63.2 (±0.3) b	101.3 (±0.30) b	15.3 (±0.4) b
DWF		11.6 (±0.1) c	59.9 (±0.4) a	64.4 (±0.3) a	102.6 (±0.32) a	12.9 (±0.3) c
Irrigation levels (IL)						
I_10D_		12.3 (±0.2) b	59.0 (±0.5) ab	63.5 (±0.4) ab	101.6 (±0.50) ab	14.5 (±0.5) bc
I_40_		11.6 (±0.1) c	59.5 (±0.4) a	64.0 (±0.3) a	102.1 (±0.35) a	13.7 (±0.5) c
I_50_		12.0 (±0.1) bc	58.4 (±0.4) b	62.9 (±0.3) b	101.0 (±0.39) b	15.4 (±0.5) ab
I_60_		12.9 (±0.2) a	57.2 (±0.5) c	61.7 (±0.4) c	99.7 (±0.48) c	15.8 (±0.4) a
Year						
2015		12.0 (±0.1) b	60.0 (±0.2) a	64.0 (±0.2) a	102.3 (±0.27) a	15.9 (±0.5) a
2016		12.4 (±0.07) a	57.1 (±0.2) b	62.1 (±0.2) b	100.2 (±0.27) b	13.7 (±0.4) b
Source of variation	DF	^__________________________________^*p*-value (<0.05)^____________________________________________________________^				
PM	2	<0.0001	<0.0001	<0.0001	<0.0001	<0.0001
IL	3	<0.0001	<0.0001	<0.0001	<0.0001	<0.0001
Year	1	0.0009	<0.0001	<0.0001	<0.0001	<0.0001
PM × IL	6	NS	NS	NS	NS	NS
PM × IL × Year	6	NS	NS	NS	NS	NS

* BBF, broad bed and furrow; SNF, shallow and narrow furrow; DWF, deep and wider furrow; I_10D_, irrigation once in 10 days; I_40_, irrigation at 40% DASM; I_50_, irrigation at 50% DASM; and I_60_, irrigation at 60% DASM. ** Standard error of mean. *** Means followed by the same letter (s) within a column are not significantly different.

**Table 7:** Average seasonal rainfall, maximum and minimum air temperatures, relative humidity, evaporation, and soil temperature of the experimental site at Dharwad in 2015 and 2016.

Month	Rainfall (mm)		Maximum Temperature (°C)		Minimum Temperature (°C)		Relative Humidity (%)		Evaporation (mm day^−1^)		Soil Temperature at 5 cm Depth (°C)	
	2015	2016	2015	2016	2015	2016	2015	2016	2015	2016	2015	2016
February	0.0	0.2	31.8	33.6	14.6	17.9	40.0	62.4	6.0	6.1	36.0	38.2
March	105.2	2.4	33.2	36.1	19.3	20.6	55.0	59.6	5.5	6.8	36.1	40.2
April	13.2	20.4	35.1	38.0	20.3	21.6	51.0	73.2	6.2	8.5	42.0	44.4
May	129.4	82.8	34.7	36.0	21.9	22.1	63.0	80.7	6.0	8.3	36.4	40.8

**Table 8:** Effect of planting methods, irrigation levels and year on 100 grain weight, cob weight, grain yield and biomass WUE of maize at Dharwad, India.

Treatment *		100 Grain Weight (g)	Cob Weight (g cob^−1^)	Grain Yield (kg ha^−1^)	Biomass WUE (kg ha-mm^−1^)
Year					
2015		29.8 (±0.1 **) a	177.9 (±0.8) a	6903 (±118.6) a	34.2 (±0.2) a***
2016		29.1 (±0.1) b	172.5 (±0.8) b	6465 (±103.8) b	31.7 (±0.3) b
P × I					
BBF	I_10D_	27.5 (±0.4) de	163.1 (±3.51) e	5907 (±94.98) de	35.8 (±1.1) bc
	I_40_	28.9 (±0.4) b–d	173.9 (±2.11) cd	6535 (±210.7) bc	33.7 (±0.2) c–e
	I_50_	29.9 (±0.5) cd	170.5 (±1.21) de	6358 (±114.7) c	37.8 (±0.4) b
	I_60_	26.0 (±0.2) e	148.0 (±2.13) f	5551 (±122.3) e	42.5 (±0.7) a
SNF	I_10D_	29.0 (±0.3) b–d	176.8 (±1.64) b–d	6427 (±84.8) bc	32.1 (±0.6) d–f
	I_40_	29.7 (±0.2) bc	183.7 (±1.65) ab	7386 (±128.1) a	29.6 (±0.5) g
	I_50_	30.5 (±0.4) ab	181.4 (±1.17) a–c	7573 (±178.7) a	34.0 (±0.6) cd
	I_60_	29.5 (±0.3) bc	168.2 (±3.90) de	6210 (±93.7) cd	35.9 (±0.8) bc
DWF	I_10D_	30.7 (±0.5) ab	183.4 (±2.11) a–c	6820 (±141.5) b	27.0 (±0.8) h
	I_40_	32.0 (±0.4) a	188.6 (±1.83) a	7335 (±64.9) a	26.9 (±0.7) h
	I_50_	31.8 (±0.4) a	189.6 (±1.80) a	7512 (±121.9) a	29.9 (±0.9) fg
	I_60_	29.7 (±0.3) bc	175.4 (±1.54) b–d	6594 (±172.0) bc	31.5 (±1.0) e–g
Source of variation	DF	^__________________________________^*p*-value (<0.05)^_____________________________________^			
PM	2	<0.0001	<0.0001	<0.0001	<0.0001
IL	3	<0.0001	<0.0001	<0.0001	<0.0001
Year	1	0.002	<0.0001	<0.0001	<0.0001
PM × IL	6	0.030	0.065	0.004	0.005
PM × IL × Year	6	NS	NS	NS	NS

* PM, planting methods; IL, irrigation levels; BBF, broad bed and furrow; SNF, shallow and narrow furrow; DWF, deep and wider furrow; I_10D_, irrigation once in 10 days; I_40_, irrigation at 40% DASM; I_50_, irrigation at 50% DASM; and I_60_, irrigation at 60% DASM. ** Standard error of mean. *** Means followed by the same letter (s) within a column are not significantly different.

**Table 9:** Effect of planting methods and irrigation levels on total water application and number of irrigations in maize.

Treatment *		Total Water Applied (mm)	Number of Irrigations
Year			
2015		439.0 (±0.3 **) b	5.8 (±0.1) b ***
2016		495.6 (±0.3) a	7.6 (±0.1) a
*p*-value (<0.05)		<0.0001	<0.0001
PM × IL			
BBF	I_10D_	372.0 (±2.7) h	6.0 (±0.5) f
	I_40_	415.5 (±2.4) f	7.0 (±0.6) d
	I_50_	372.0 (±2.7) h	6.0 (±0.5) f
	I_60_	328.5 (±2.4) i	5.0 (±0.6) g
SNF	I_10D_	453.5 (±8.7) e	6.5 (±0.8) e
	I_40_	510.5 (±8.7) c	8.0 (±0.5) b
	I_50_	453.5 (±8.7) e	7.0 (±0.5) d
	I_60_	396.5 (±8.7) g	6.0 (±0.6) f
DWF	I_10D_	552.5 (±15.8) b	6.5 (±0.4) e
	I_40_	625.5 (±15.8) a	8.7 (±0.5) a
	I_50_	552.5 (±15.9) b	7.0 (±0.4) d
	I_60_	479.0 (±16.1) d	6.5 (±0.4) e
*p*-value (<0.05)		<0.0001	<0.0001

* PM, planting methods; IL, irrigation levels; BBF, broad bed and furrow; SNF, shallow and narrow furrow; DWF, deep and wider furrow; I_10D_, irrigation once in 10 days; I_40_, irrigation at 40% DASM; I_50_, irrigation at 50% DASM; and I_60_, irrigation at 60% DASM. ** Standard error of mean. *** Means followed by the same letter (s) within a column are not significantly different.

**Table 10:** Selected soil physico-chemical properties of the experimental site.

Soil Layers (cm)	Bulk Density (g c^−3^)	Porosity (%)	Soil Texture	Soil Particle Fraction (%)			Field Capacity (%)	Permanent Wilting Point (%)
				Sand (>0.05 mm)	Silt (0.05–0.002 mm)	Clay (<0.002 mm)		
0–15	1.2	54.2	Clayey	18.8	33.4	47.2	32.4	18.0
15–30	1.3	52.4	Clayey	21.0	32.1	46.8	32.7	18.1
30–45	1.3	52.4	Clayey	12.1	34.2	53.6	33.9	18.1

## Data Availability

All data are available in this publication.

## Abbreviations

BBF	broad bed and furrow
CGR	crop growth rate
DAS	days after sowing
DASM	depletion of available soil moisture
DWF	deep and wider furrow
LAD	leaf area duration
SNF	shallow and narrow furrow
SPAD	soil plant analysis and development
RWC	relative water content
WUE	water use efficiency
